# Human Mesenchymal Stem Cells Prevent Neurological Complications of Radiotherapy

**DOI:** 10.3389/fncel.2019.00204

**Published:** 2019-05-16

**Authors:** Bernat Soria, Alejandro Martin-Montalvo, Yolanda Aguilera, Nuria Mellado-Damas, Javier López-Beas, Isabel Herrera-Herrera, Escarlata López, Juan A. Barcia, Manuel Alvarez-Dolado, Abdelkrim Hmadcha, Vivian Capilla-González

**Affiliations:** ^1^Department of Regeneration and Cell Therapy, Andalusian Center for Molecular Biology and Regenerative Medicine (CABIMER), University of Pablo de Olavide – University of Seville, CSIC, Seville, Spain; ^2^Centro de Investigación Biomédica en Red de Diabetes y Enfermedades Metabólicas Asociadas (CIBERDEM), Madrid, Spain; ^3^Department of Neuroradiology, Hospital Universitario Fundación Jiménez Díaz, Madrid, Spain; ^4^Department of Radiation Oncology, Hospital Universitario Fundación Jiménez Díaz, Madrid, Spain; ^5^Service of Neurosurgery, Hospital Clínico San Carlos, Instituto de Investigación Sanitaria San Carlos (IdISSC), Department of Surgery, Universidad Complutense de Madrid, Madrid, Spain

**Keywords:** radiotherapy, stem cells, cognition, neuroprotection, intranasal cell delivery, CREB, neurocognitive sequelae, brain cancer

## Abstract

Radiotherapy is a highly effective tool for the treatment of brain cancer. However, radiation also causes detrimental effects in the healthy tissue, leading to neurocognitive sequelae that compromise the quality of life of brain cancer patients. Despite the recognition of this serious complication, no satisfactory solutions exist at present. Here we investigated the effects of intranasal administration of human mesenchymal stem cells (hMSCs) as a neuroprotective strategy for cranial radiation in mice. Our results demonstrated that intranasally delivered hMSCs promote radiation-induced brain injury repair, improving neurological function. This intervention confers protection against inflammation, oxidative stress, and neuronal loss. hMSC administration reduces persistent activation of damage-induced c-AMP response element-binding signaling in irradiated brains. Furthermore, hMSC treatment did not compromise the survival of glioma-bearing mice. Our findings encourage the therapeutic use of hMSCs as a non-invasive approach to prevent neurological complications of radiotherapy, improving the quality of life of brain tumor patients.

## Introduction

Radiotherapy is one of the most common treatments for cancer. Around 50% of all tumor patients receive radiation at a given time (Delaney et al., 2005). Unfortunately, radiotherapy comes with short and long term side effects. In particular, radiation for brain tumors, the most common cancer in children (Ostrom et al., 2016), causes accelerated aging that is manifested as neurofunctional sequelae, which may be progressive and permanent (Douw et al., 2009; Marazziti et al., 2012; Padovani et al., 2012; Armstrong et al., 2013; Ma et al., 2017). The most frequently described adverse effects of cranial radiation include learning and memory difficulties, problems in executive functions, motor coordination, visual alterations and intellectual decline. These radiation-related sequelae compromise the quality of life of cancer survivors and represent a serious clinical problem with no satisfactory solutions at present.

Studies in brain cancer patients and rodent evidences that radiation-related neurofunctional sequelae are associated with a variety of anatomical changes that occur in the irradiated non-tumoral tissue (Makale et al., 2017). Immediately after radiation, brain exhibits vascular damages, oligodendrocyte loss, demyelination and neuroinflammation. Radiation-induced brain injury also disrupts the neurogenic niches located at the dentate gyrus (DG) of the hippocampus and the subventricular zone (SVZ) of the lateral ventricles. Moreover, brain injury also affects neuronal dendritic spines and white matter, leading to necrosis of specific areas. The discovery of the negative effects induced by radiation in the non-tumoral tissue has promoted the development of strategies to minimize radiotherapy side effects. In this context, stem cell-based therapy represents a novel alternative to attenuate radiation-induced brain injury (Acharya et al., 2011, 2015; Joo et al., 2012; Piao et al., 2015). In this line, Joo et al. (2012) described the benefits of supplementing whole-brain irradiated mice with fetal mouse neural stem cells (NSCs), which were injected via tail vein 24 h after radiation. The irradiated brain induced homing of the exogenous NSCs, which differentiated along glial and neuronal lineages. Two months after NSC administration, mice showed inhibited radiation-induced hippocampus atrophy and preserved short-term memory. Similarly, human embryonic stem cell-derived oligodendrocyte progenitors (hOPCs) have provided promising results. After bilateral injections into the corpus callosum of rats, hOPCs were able to remyelinate the brain and ameliorate radiation-induced cognitive dysfunction (Piao et al., 2015). However, stem cell-based therapies proposed in current studies present some restrictions that need to be solved if translation to human is sought (Ramos-Zuriga et al., 2012). First, several studies used stem cells with scarce availability and whose isolation procedure is highly invasive (e.g., NSCs). Second, the routes used for administration have limited effectiveness (e.g., systemic transplantation renders reduced concentration of transplanted cells in the brain) or requires invasive techniques that risk host safety (e.g., intracranial injections). Here we explored the non-invasive intranasal delivery of human mesenchymal stem cells (hMSCs) derived from adipose tissue to prevent radiation-induced brain damage in a mouse model of whole-brain radiation. Our results demonstrated that transplanted hMSCs promoted neuroprotection and improved neurological function after irradiation, without compromising survival of glioma-bearing mice.

## Materials and Methods

### Animals

Two-month-old male C57BL/6 mice were purchased from Charles River Laboratories (Barcelona, Spain). For cell transplant, two-month-old male immunodeficient athymic nude mice (Charles River Laboratories) were used to maximize the non-rejection and survival of transplanted cells. Experimental groups were randomly assigned. Mice were housed in a specific pathogen free animal facility. Animals were maintained on a 12-h light/dark cycle, with stable temperature (22°C) and humidity (60%), and with food and water available *ad libitum*.

### X-Ray Irradiation

Mice were anesthetized via intraperitoneal injection of a combination of 100 mg/kg ketamine and 10 mg/kg xylazine. Then, animals were positioned in a prone position in the X-ray irradiation device (MBR-1505R; Hitachi, Tokyo, Japan) for head-only irradiation, as described elsewhere (Suarez-Pereira et al., 2015). Animals were irradiated at 160 kV and 6.3 mA with a lead shield covering the entire body, excluding the head. A total dose of 10 Gy in 2 fractions (2 × 5 Gy) was delivered at a source-to-skin distance of 33 cm. Control animals were littermates handled similarly and did not receive radiation.

### hMSC Culture

Human mesenchymal stem cell (ATCC, PCS-500-011^TM^; see Supplementary Table 1 for cell lines information) were cultured in growth media composed of Dulbecco’s Modified Eagle Medium (DMEM; Life Technologies, Carlsbad, CA) supplemented with 10% fetal bovine sera and 1% penicillin-streptomycin, and incubated at 37°C in a 20% O_2_ and 5% CO_2_ humidified atmosphere. Media were changed every 2–3 days. For all experiments, hMSCs were used at passage 4–7. The ability of hMSCs to generate multiple lineages and express established MSC markers was previously verified (Capilla-Gonzalez et al., 2018).

### hMSC Transplantation and Biodistribution

Human mesenchymal stem cell treatment was initiated the day after radiation. Briefly, animals were anesthetized and placed in a supine position to administrate total of 100 U of hyaluronidase as 2 repeated inoculations in each nostril with 5-min intervals (3 μl per inoculation). After 30 min, 5 × 10^5^ of hMSCs in PBS were delivered as 2 repeated inoculations in each nostril with 5-min intervals (3 μl per inoculation). Mice received a dose of cells per week during 4 consecutive weeks. Control mice received hyaluronidase followed by PBS. For evaluation of cell biodistribution, cultured hMSCs were incubated with 400 μg/mL XenoLight DiR fluorescent dye (Perkin Elmer, Inc., Boston, MA) for 30 min at 37°C before transplantation. Transplanted mice were daily monitored using an IVIS Imaging System 200 Series (Caliper Life Science, Hopkinton, MA).

### Behavioral Tests

Neurological function was tested between day 33 and day 44 post-radiation using a battery of behavioral tests, following previously described protocols. First, motor coordination was evaluated by *rotarod* performance (Lopez-Noriega et al., 2017) on days 33–34. Second, muscle strength was evaluated by the *wirehang* test (Klein et al., 2012) on day 35. Third, olfaction was evaluated by measuring odor discrimination capacity in a two-odorants test (*habituation-dishabituation test*) (Capilla-Gonzalez et al., 2012) on day 37. Finally, cognition was assessed by performing the *novel object recognition* task with a long habituation phase, using odorless objects that do not retain any olfactory cues (Leger et al., 2013), on days 40–44.

### Microarray

RNA was isolated from the brain lateral ventricle (PLv) and the hippocampus (Hipp) using the RNeasy Mini Kit (Qiagen, Hilden, Germany). 100 ng of RNA was used to obtain the gene expression profile of each sample. All samples showed the characteristics of high-quality RNA and were subjected to subsequent analysis. cDNA was hybridized to the Clariom^TM^ S Assay Mouse Array (Affymetrix, Santa Clara, CA) using manufacturer’s protocol (Affymetrix, GeneChip WT PLUS). Microarrays were scanned using the GeneChip Scanner 3000 7G of Affymetrix. Data processing and statistical analysis was performed using Transcriptome Analysis Console (TAC) software from Affymetrix, using default parameters. Canonical pathway analysis was performed using Ingenuity Pathway Analysis (IPA) software from Qiagen. Venn diagrams were generated using the open-source online tool Venny 2.1.0. Microarray data are deposited in Gene Expression Omnibus (GEO) database repository (accession number: GSE115735).

### Western Blots

Dissected PLv and Hipp were lysed for protein extraction using RIPA buffer (Sigma-Aldrich, Madrid, Spain), containing 0.5% sodium deoxycholate, 1 mM PMSF, 2 mM EDTA, 1× protease inhibitor (Roche Diagnostics, Mannheim, Germany) and 1× phosphatase inhibitor cocktail (Thermo Fisher Scientific, Madrid, Spain). Proteins from whole tissue lysates (25 μg) were resolved using 10% Tris-Glycine gel electrophoresis, and transferred onto nitrocellulose membranes (Whatman, Dassel, Germany). Membranes were then blocked with 5% non-fat milk and primary antibodies were probed (see Supplementary Table 1 for antibody information). Detection was done with the appropriate horseradish-peroxidase conjugated secondary antibodies and using the enhanced chemiluminescence reagent (GE Healthcare Life Sciences, Buckinghamshire, United Kingdom). Densitometric analyses for the blots were performed using ImageJ software (version 1.4r; National Institute of Health, Bethesda, MD) and normalized to Ponceau S staining or GAPDH expression.

### Brain Tissue Fixation

Mice were anesthetized and subjected to intracardiac perfusion using a peristaltic pump. As a fixative, 2% paraformaldehyde and 2.5% glutaraldehyde was used for electron microscopy, while 4% paraformaldehyde was used for immunohistochemistry. Brains were removed and post-fixed in the same fixative solution overnight.

### Transmission Electron Microscopy

Fixed brains were rinsed in 0.1 M phosphate buffer (PB) and cut into 200 μm sections using a VT 1000M vibratome (Leica, Wetzlar, Germany). Sections were postfixed in 2% osmium tetroxide, dehydrated in ethanol, stained in 2% uranyl acetate and embedded in araldite (Durcupan, Fluka BioChemika, Ronkokoma, NY). Ultrathin sections (60–70 nm) were cut with a diamond knife, stained with lead citrate, and examined under a Spirit transmission electron microscope (FEI Tecnai, Hillsboro, OR) (Capilla-Gonzalez et al., 2010).

### Immunohistochemistry

Fixed brains were rinsed in 0.1 M phosphate buffer (PB) and cut into serial 10 μm thick sections using a CM 3050S cryostat (Leica, Mannheim, Germany). Sections were incubated in blocking solution for 1 h at room temperature, followed by overnight incubation at 4°C with primary antibodies (see Supplementary Table 1 for antibody information). Then, sections were washed and incubated with the appropriate secondary antibodies conjugated with fluorophores and examined under a Leica DM6000B microscope or Leica TC5 SP5 confocal microscope and imaged with the Leica Application Suite software. Fluorescence signal was quantified using ImageJ or MetaMorph software (Molecular Devices, San Jose, CA).

### Brain Tumor Model

To investigate the side effects effect of transplanting hMSCs into mice bearing a brain tumor, 7-weeks-old male immunodeficient athymic nude mice were stereotactically injected with 0.5 × 10^6^ glioma cells (U87MG; ATCC, HTB-14^TM^; see Supplementary Table 1 for cell lines information) into the right striatum (A: 0.5, L: 2.0, D: 3.5). The stereotactic surgery procedure was done as previously described (Capilla-Gonzalez et al., 2014). Prior transplantation, glioma cells were labelled with the XenoLight DiR fluorescent dye, following the same protocol described for hMSCs labeling. The days after cell transplant, mice were imaged using an IVIS Imaging System 200 Series (Caliper Life Science, Hopkinton, MA) to ensure that grafted cells form a tumor. Ten days after tumor cell injection, animals were randomly distributed into three groups: mice bearing a brain tumor (*n* = 11), mice bearing a brain tumor that received radiation (*n* = 11), and mice bearing a brain tumor that received radiation and hMSCs (*n* = 12). Radiation and hMSC transplant were given as described above. Mice were euthanized when the condition of the animal was considered incompatible with continued survival. Survival curves were plotted using the Kaplan–Meier method, which include any animal found dead or euthanized. Histopathological analysis of brains at time of death was performed on cryosections stained with hematoxylin and eosin method.

### Statistical Analysis

Data were expressed as mean ± SEM. Data were analyzed using the GraphPad Prism 7 software (GraphPad Software Inc., San Diego, CA) and SigmaPlot 12.0 software (Jandel Scientific, San Rafael, CA). The *t*-test was performed to compare two means, while ANOVA was performed to compare more than two means. Repeated-measures ANOVA and Multiple *t*-test were applied when appropriated. Log-rank test was performed to determine differences in survival. All differences were considered significant at a *p* value <0.05.

## Results

### Radiation Induces Anatomical Changes in the Mouse Brain

**FIGURE 1:**
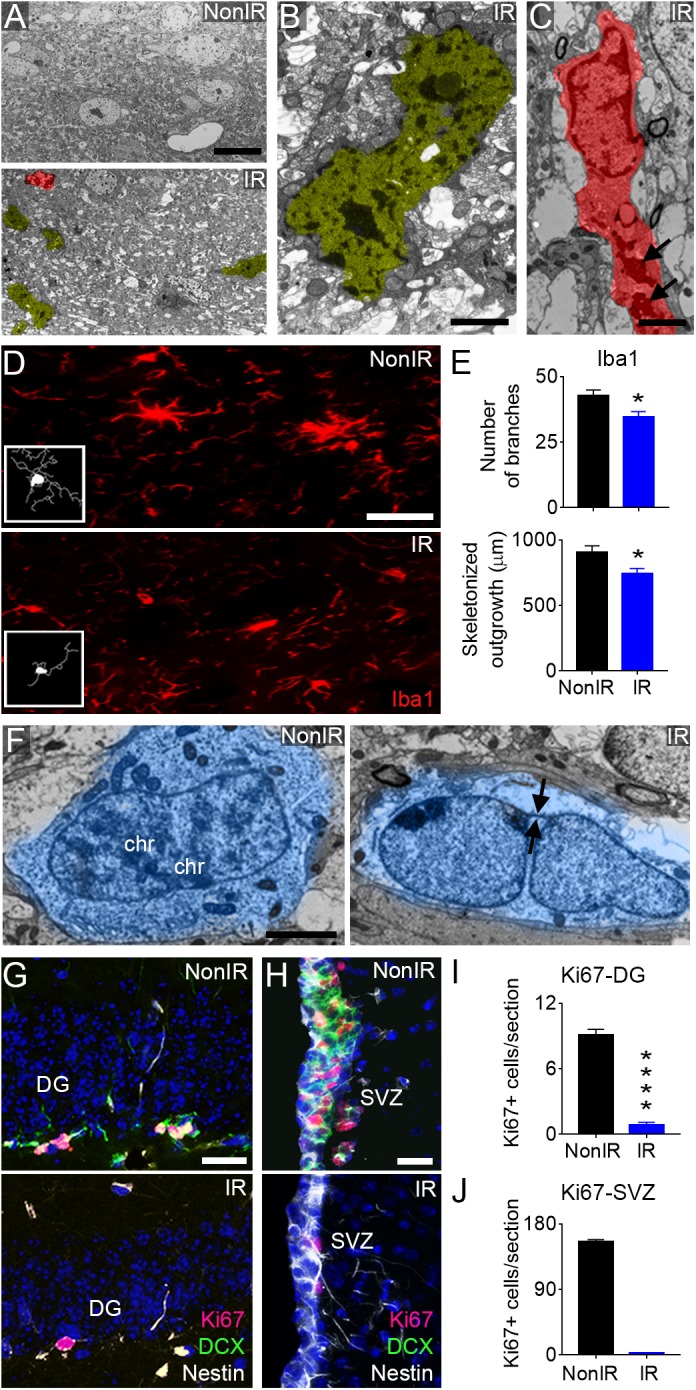
Radiation induces anatomical changes in the mouse brain. Representative images of radiation-induced damages in the mouse brain 30 days post-radiation (total dose of 10 Gy). **(A)** Electron microscopy micrograph showing multiple pyknotic cells (yellow) and microglia (red) in the irradiated brain, compared to non-irradiated mice. **(B)** High magnification of a pyknotic cell in the irradiated brain. **(C)** High magnification of a microglia cell in the irradiated brain displaying round shape and abundant dense bodies (arrows) in the cytoplasm. **(D)** Immunofluorescence against Iba1 reveals the presence of ramified microglia (resting cells) in the non-irradiated brain and bushy microglia (activated cells) in the irradiated brain. **(E)** Quantification of the number of branches and total length of processes (skeletonized outgrowth) in Iba1 expressing cells. **(F)** Representative images showing the presence of a NSC with condensed chromosomes (chr) in the non-irradiated SVZ and a quiescent NSC identified by the presence of envelope-limited chromatin sheets (arrows) in the irradiated SVZ. **(G)** Immunofluorescence against Ki67, Nestin, and DCX in the irradiated DG. **(H)** Immunofluorescence against Ki67, Nestin, and DCX in the irradiated SVZ. **(I)** Quantification of Ki67+ cells in the DG. **(J)** Quantification of Ki67+ cells in the SVZ. IR, irradiated mice; NonIR, non-irradiated mice. Scale bar: **(A)** 10 μm, **(B,C)** 2 μm, **(D)** 50 μm, **(F)** 2 μm, **(G)** 25 μm, **(H)** 200 μm. Data are represented as mean ± SEM. *n* = 5 per group, ^∗^*p* < 0.05, ^∗∗∗∗^*p* < 0.0001; *t*-test.

To characterize the impact of radiation in mouse brain, two month-old C57BL/6 mice were subjected to whole-brain radiation (total dose of 10 Gy). The histopathological analysis of mouse brain tissue by electron microscopy revealed frequent necrotic cells and microglia 30 days post-radiation (Figure 1A–C). Microglia staining to detect Iba1 expression evidenced bushy microglia (activated cells) in the irradiated brain that manifested an inflammatory state, while the non-irradiated brain displayed more ramified microglia (resting cells) (Figure 1D–E). Furthermore, NSCs residing in the irradiated brain frequently presented envelope-limited chromatin sheets (i.e., quiescent NSCs), while NSCs in the non-irradiated brain often displayed condensed chromosomes associated with an early prophase (i.e., active NSCs) (Figure 1F). This observation was in line with the reduced proliferation (Ki67 staining) and scarce newly born neurons (DCX, doublecortin staining) in the DG and SVZ after radiation, while the NSC marker nestin showed no evident differences (Figure 1G–J).

### hMSCs Reached the Brain After Intranasal Administration

**FIGURE 2:**
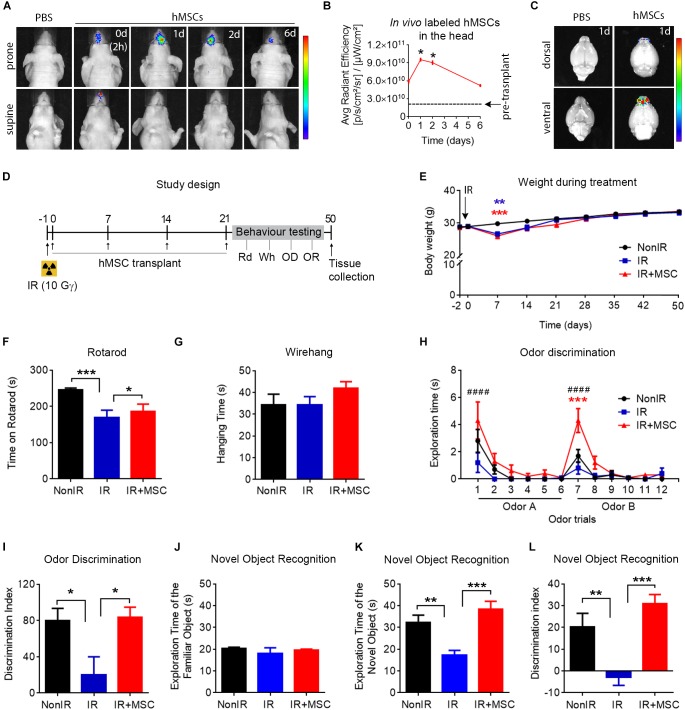
Transplanted hMSCs improved neurological function after whole-brain radiation. **(A)** Representative images showing *in vivo* fluorescence signal in the head at 2 h, 1 day, 2 days, and 6 days after the first intranasal cell delivery (5 × 10^5^ hMSCs). **(B)** Quantification of the *in vivo* fluorescence signal in the head within the first 6 days post transplantation, compared to day 0 (2 h after hMSC delivery). **(C)** *In vivo* fluorescence signal in dissected brains 1 day after intranasal delivery of hMSCs. **(D)** Schematic representation of the study design. Mice received whole-brain radiation (total dose of 10 Gy). Twenty-four hours after radiation, 5 × 10^5^ cells hMSCs were administered intranasally once per week for 4 weeks. Then, mice were tested in a behavioral test battery (Rotarod, Rd on days 33–34; Wirehang, Wh on day 35; Odor discrimination task, OD on day 37; Novel Object Recognition test, OR on days 40–44) before being sacrificed. **(E)** Body weight during the course of the experiment revealing a temporary weight loss in irradiated mice (receiving or not hMSCs) the week after radiation exposure. **(F)** Rotarod test performance showing an improvement in IR+MSC mice, as compared to IR mice. **(G)** Wirehang test performance showing no differences between any experimental group. **(H)** Time spent sniffing the stimuli (Odor A and Odor B) in an odor discrimination task. **(I)** Discrimination index of Odor A and Odor B (presentation 6 vs. presentation 7), showing a rescued OD performance in IR+MSC mice. **(J)** Exploration time of the familiar object in the Novel Object Recognition test. **(K)** Exploration time of the novel object in the Novel Object Recognition test. **(L)** Discrimination index between familiar and novel object, showing a rescued OR performance in IR+MSC mice. Data are represented as mean ± SEM. *n* = 3 per group **(B)**, *n* = 10–15 per group **(E–L)**. Comparisons of IR or IR+MSC versus Non-IR are indicated with ^∗^. Comparisons of IR+MSC versus IR are indicated with ^#^. ^∗^*p* < 0.05, ^∗∗^*p* < 0.01, ^∗∗∗^*p* < 0.001, ^∗∗∗∗^*p* < 0.0001, ^####^*p* < 0.0001; One-way ANOVA **(B,F,G,I–L)**, Two-way repeated-measures ANOVA **(E,H)**. Rainbow color scale: red indicates highest fluorescence signal and blue indicates lowest fluorescence signal.

To validate that the intranasal route is a feasible via to deliver cells to the brain, XenoLight DiR-labeled hMSCs were administrated into the nostrils of athymic nude mice (a dose of cells per week during 4 consecutive weeks) (Supplementary Figure 1). A cohort of animals was injected with PBS as control group. Biodistribution analysis revealed that 2 h after the first dose of cells, fluorescence was restricted to the head, becoming maximal at day 1 and then tending to gradually decrease within the following 6 days (Figure 2A,B). However, the repeated doses of cells presented a cumulative effect that allowed to prolong fluorescence signal over time (Supplementary Figure 1). Temporary fluorescence signal was observed in the peritoneal region 24 h after cell delivery (Supplementary Figure 1). No signal was observed in mice treated with PBS at any time point. In order to demonstrate the presence of hMSCs in the brain, a group of mice was sacrificed the day after cell administration and brains were dissected. Fluorescence signal was detected in the olfactory bulbs and frontal lobes (Figure 2C). To analyze whether hMSCs persist into the brain on day 50, immunofluorescence against human-specific mitochondria (hMito) and human-specific nuclei (hNuclei) was performed in brain sections. Immunostaining revealed the presence of some human cells at different levels of the rostrocaudal brain axis (Supplementary Figure 1). Necropsies did not reveal any visible tumor mass in mice transplanted with hMSCs 50 days after the first cell dose. These results indicate that intranasal delivery is a feasible route to administrate cells to the brain.

### Transplanted hMSCs Improved Neurological Function After Whole-Brain Radiation

We then examined whether intranasally delivered hMSCs could ameliorate neurological function in whole-brain irradiated mice. For this, animals were randomly assigned to three experimental groups: Non-irradiated mice receiving PBS (NonIR), irradiated mice receiving PBS (IR) and irradiated mice receiving hMSCs (IR+MSC). hMSC treatment (a dose of cells per week during 4 consecutive weeks) was initiated 24 h after radiation (Figure 2D). First, body weight was monitored during the course of the experiment (Figure 2E) as indicative of animal health status. We found a significant weight loss the week after radiation in IR and IR+MSC mice that was rapidly recovered, reaching the values of NonIR animal by 2-weeks post-irradiation. Then, animals were subjected to serial behavior testing to evaluate motor coordination (rotarod), muscle strength (wirehang), olfaction (odor discrimination task; OD) and cognition (novel object recognition test; OR). The IR group showed poor rotarod performance, as compared to NonIR and IR+MSC mice (Figure 2F). Wirehang performance was not different between any experimental group, indicating that results from rotarod were not influenced by muscle strength (Figure 2G). Then, mice were subjected to OD test. IR mice showed impaired ability to discriminate between two odorants, as compared to the Non-IR group, while hMSC treatment rescued OD performance (Figure 2H,I). Finally, mice were tested to the OR task. Exploration time of the familiar object was not different between any experimental groups, while exploration time of the novel object was reduced in IR mice, as compared to the other groups (Figure 2J,K). The discrimination index indicated that both, NonIR and IR+MSC mice exhibited a similar preference for the novel object (Figure 2L). These observations demonstrated a robust effect of hMSC treatment on neurofunctional recovery after radiation.

### The Gene Expression Profile of the Irradiated Brain Was Modulated by hMSC Transplantation

**FIGURE 3:**
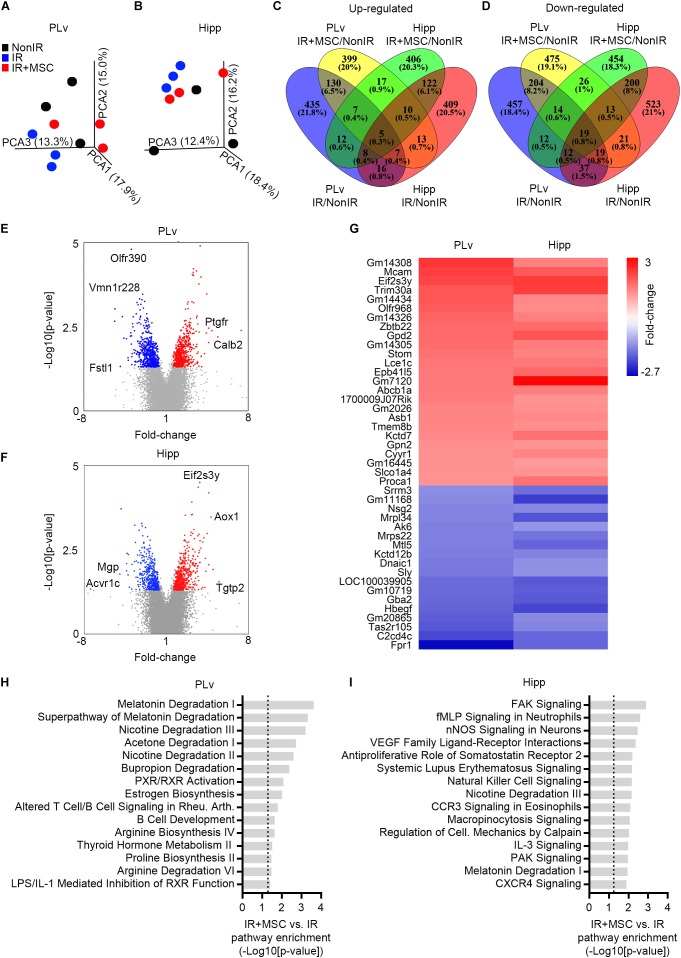
The gene expression profile of the irradiated brains was modulated by hMSC transplantation. **(A)** PCA of genes expressed in the PLv. Plots represent individual samples. **(B)** PCA of genes expressed in the Hipp. Plots represent individual samples. **(C)** Venn diagrams showing the number and percentage of significantly up-regulated genes between the different tissues (PLv and Hipp) and different experimental groups (IR vs. NonIR and IR+MSC vs. NonIR). **(D)** Venn diagrams showing the number and percentage of significantly down-regulated genes between the different tissues (PLv and Hipp) and different experimental groups (IR vs. NonIR and IR+MSC vs. NonIR). **(E)** Volcano plot showing the fold change and statistical significance of genes expressed in the comparison IR+MSC vs. IR in the PLv. **(F)** Volcano plot showing the fold change and statistical significance of genes expressed in the comparison IR+MSC vs. IR in the Hipp. **(G)** Heat map depicting relative expression levels of shared significantly modulated genes in IR+MSC as compared to IR in PLv and Hipp. **(H)** Comparison of canonical pathways significantly altered in the PLv of IR+MSC as compared to IR using the IPA platform. **(I)** Comparison of canonical pathways significantly altered in the Hipp of IR+MSC as compared to IR using the IPA platform. *n* = 3 per group.

After behavioral testing (i.e., at day 50 post-radiation; Figure 2D), all mice were sacrificed and brain tissue was collected to evaluate the molecular and cellular changes induced by hMSC administration. First, a group of mice was used to characterize genome-wide gene expression modulations in the PLv and Hipp, which contain the neurogenic niches. Principal component analysis (PCA) showed a distinct global transcriptional profile in the PLv and Hipp of IR mice, as compared to NonRx animals, while it was less evident in samples of animals receiving hMSCs (Figure 3A,B). Gene-expression profiling revealed that the vast majority of the genes whose expression was significantly up-regulated (∼81.3%) or down-regulated (∼76.8%) by radiation or radiation plus hMSCs in each tissue were specific to the tissue and experimental condition, indicating an overall distinct expression profile (Figure 3C,D). The analysis of genes significantly modulated in the comparison IR+MSC versus IR indicated that 1210 genes were differentially expressed in the PLv and 1050 in the Hipp (Figure 3E,F and Supplementary Tables 2, 3). Of these, Calretinin (Calb2), a calcium-binding protein that plays a role in neuron excitability, was among the most up-regulated transcripts in the PLv of IR+MSC mice. The T cell specific GTPase 2 (Tgtp2), a member of the immune-related p47 GTPases, and the aldehyde oxidase 1 (Aox1), involved in Phase I metabolism of xenobiotics, were among the most highly over-expressed genes in the Hipp of IR+MSC mice. Several genes were similarly regulated in the PLv and Hipp of IR+MSC mice when compared to IR samples, suggesting that the modulation of these genes might contribute to the aforementioned improvements in neurological function after radiation (Figure 3G). Of these, the down-regulation of the formyl peptide receptor 1 (Fpr1) was particularly interesting since this gene is a member of the family of receptors for neutrophil chemotactic factors. Subsequent analysis using IPA indicated that several pathways involved in metabolite degradation were significantly modulated in the PLv of IR+MSC mice when compared to IR mice (Figure 3H and Supplementary Figures 2, 3). In the Hipp, the focal adhesion kinase (FAK) signaling, a key regulator of cell movement, was the most significantly modulated pathway (Figure 3I and Supplementary Figures 2, 3). Remarkably, several pathways involved in immune-related processes and melatonin degradation were altered in both, the PLv and the Hipp of IR+MSC mice when compared to IR mice (Figure 3H,I and Supplementary Figures 2, 3). A modulation of genes involved in immune response and chemotaxis was also found by Gene Ontology (GO) enrichment analysis using Database for Annotation, Visualization and Integrated Discovery (DAVID) (Supplementary Figure 4). The overall interpretation of these results is that hMSC transplantation modulates genetic pathways associated with inflammation, immune system and cell motility in mice.

### hMSC Administration Attenuates Radiation-Induced Persistent CREB Activation

**FIGURE 4:**
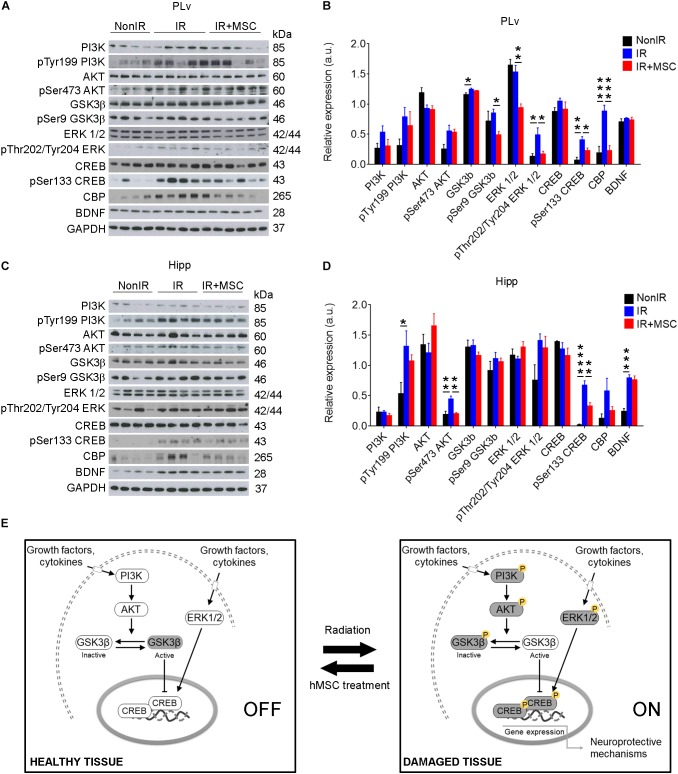
hMSC administration attenuates radiation-induced persistent CREB activation. **(A)** Western blots showing the activation of CREB signaling in the PLv of IR mice and partial normalization of CREB expression in IR+MSC mice. **(B)** Densitometric analysis of the western blots of proteins involved in CREB signaling in the PLv. Values were normalized to the GAPDH. **(C)** Western blots showing the activation of CREB signaling in the Hipp of IR mice and partial normalization of CREB expression in IR+MSC mice. **(D)** Densitometric analysis of the western blots of proteins involved in CREB signaling in the Hipp. Values were normalized to the GAPDH. **(E)** Schematic representation of proteins involved in CREB signaling modulation during radiation and hMSC treatment. Data are represented as mean ± SEM. *n* = 4–5 per group. ^∗^*p* < 0.05, ^∗∗^*p* < 0.01, ^∗∗∗^*p* < 0.001, ^∗∗∗∗^*p* < 0.0001; One-way ANOVA.

The protective effect of hMSC administration in the PLv and Hipp 50 days post-radiation (Figure 2D) was further evaluated by examining CREB signaling, a key mediator of neuroprotection (Figure 4A–D). We observed a persistent activation of the transcription factor cAMP response element-binding (CREB) through phosphorylation at Ser133 in IR mice, which mediates neuroprotection in brain injury. CREB activation was accompanied by increased expression of the CREB-binding protein (CBP), a protein required to initiate transcriptional regulations mediated by CREB. Interestingly, hMSC treatment normalized the increased levels of the active isoform of CREB (PLv: IR+MSC vs. NonIR, *p*-value = 0.0565; Hipp: IR+MSC vs. NonIR, *p*-value = 0.0065). Consistently, the PLv of IR+MSC mice exhibited lower levels of phosphorylated ERK1/2 at Thr202/Tyr204, a well-known activator of CREB, when compared to IR mice. In contrast, levels of the Ser9 phospho-inactive isoform of GSK3β, an inhibitor of CREB, were reduced in the PLv of IR+MSC mice. Interestingly, phospho-Ser9 GSK3β levels in the Hipp were similar between the different experimental groups, despite the fact that phospho-Ser473 Protein Kinase B (AKT) levels were increased in IR mice, as compared to the other groups. Furthermore, the brain-derived neurotrophic factor (BDNF), a downstream target of CREB, was up-regulated in the Hipp of mice receiving radiation. However, MSC treatment did not rescue BNDF levels. Together, these results indicate that radiation-induced damage response was down-regulated in IR+MSC mice, suggesting that hMSC administration promotes brain injury repair, as compared to IR mice (Figure 4E).

### hMSCs Protected From Neural Cell Loss, Inflammation and Oxidative Stress

**FIGURE 5:**
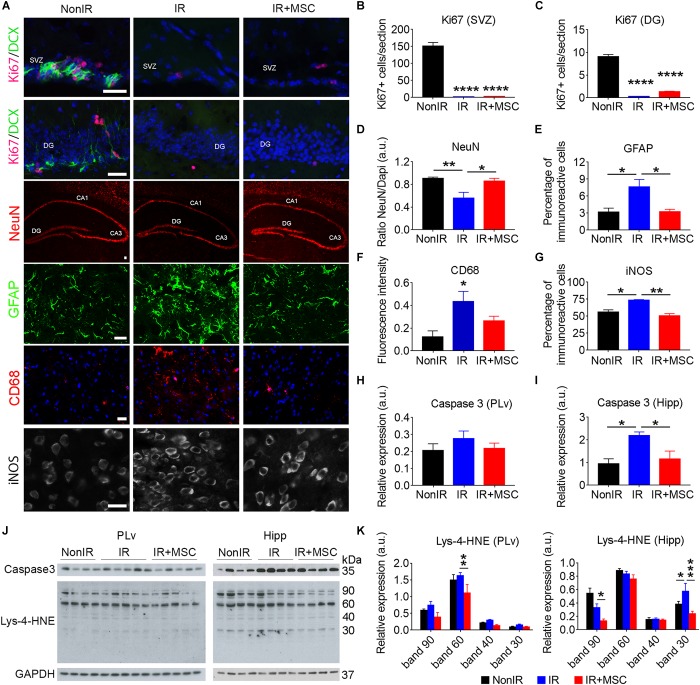
hMSCs protected from neural cell loss, inflammation and oxidative stress. **(A)** Immunofluorescence analysis in the brain of NonIR, IR and IR+MSC mice, using Ki67, DCX, NeuN, GFAP, CD68 and iNOS markers (*n* = 3–5 per group). **(B)** Quantification of Ki67 positive cells in the SVZ. **(C)** Quantification of Ki67 positive cells in the DG. **(D)** Quantification of NeuN immunoreactive area in the Hipp. **(E)** Quantification of the percentage of GFAP immunoreactivity in the striatum. **(F)** Quantification of the CD68 immunoreactivity in the cortex. **(G)** Quantification of iNOS immunoreactivity in the striatum. **(H)** Densitometric quantification of Caspase 3 expression by western blot in the PLv. Western blot images are shown in panel **(J)**. **(I)** Densitometric quantification of Caspase 3 expression by western blot in the Hipp. Western blot images are shown in panel **(J)**. **(J)** Representative western blots of Caspase 3 and Lys-4-HNE from PLv and Hipp tissue lysates (*n* = 4–5 per group). **(K)** Densitometric quantification of Lys-4-HNE levels by western blot in PLv and Hipp tissue lysates. Scale bar 25 μm. Data are represented as mean ± SEM. ^∗^*p* < 0.05, ^∗∗^*p* < 0.01, ^∗∗∗^*p* < 0.001, ^∗∗∗∗^*p* < 0.0001; One-way ANOVA **(B–I)**, Two-way ANOVA **(K)**.

To further delineate the effects of hMSC treatment in irradiated mice, we performed a brain histopathological analysis (Figure 5A). The neurogenic niches (i.e., SVZ and DG) of IR and IR+MSC mice exhibited similar levels of Ki67- and DCX-expressing cells (Figure 5B,C). However, we found that hMSC treatment preserved the number of NeuN-expressing cells in the Hipp of irradiated mice (Figure 5D). Cranial radiation significantly promoted reactive gliosis, as evidenced by a greater GFAP immunoreactivity, and induced the expression of activated microglia (CD68 marker) in IR mice, while transplanted animals exhibited expression levels near NonIR mice (Figure 5E,F). The number of cells immunoreactive for iNOS, a classic glial proinflammatory mediator, was also enhanced in the IR group, while hMSC treatment reverted this effect (Figure 5G). Plasma levels of IL-2 and IL-1β also support a normalization of the inflammatory status in transplanted mice (Supplementary Figure 5).

Protein extracts from the PLv and the Hipp were further used to evaluate the neuroprotective role of hMSC infusion. We found that hMSC treatment prevented the elevation of radiation-induced Caspase 3 expression in the Hipp by western blot. In contrast, the expression of Caspase 3 in the PLv did not show differences across the experimental groups (Figure 5H–J). We determined the oxidative damage by quantifying the levels of lysine-4-hydroxynonenal (Lys-4-HNE), a marker of protein-bound lipid peroxidation. The irradiated PLv displayed increased levels of Lys-4-HNE that were reduced in the PLv of IR+MSC mice. The reduction of Lys-4-HNE levels in the Hipp of IR+MSC was less robust (Figure 5J,K). These data suggest that hMSC administration reduces neuroinflammation, oxidative damage and neuronal loss in whole-brain irradiated mice, although it does not prevent neurogenesis decline.

### hMSC Administration Does Not Compromise Survival of Glioma-Bearing Mice

**FIGURE 6:**
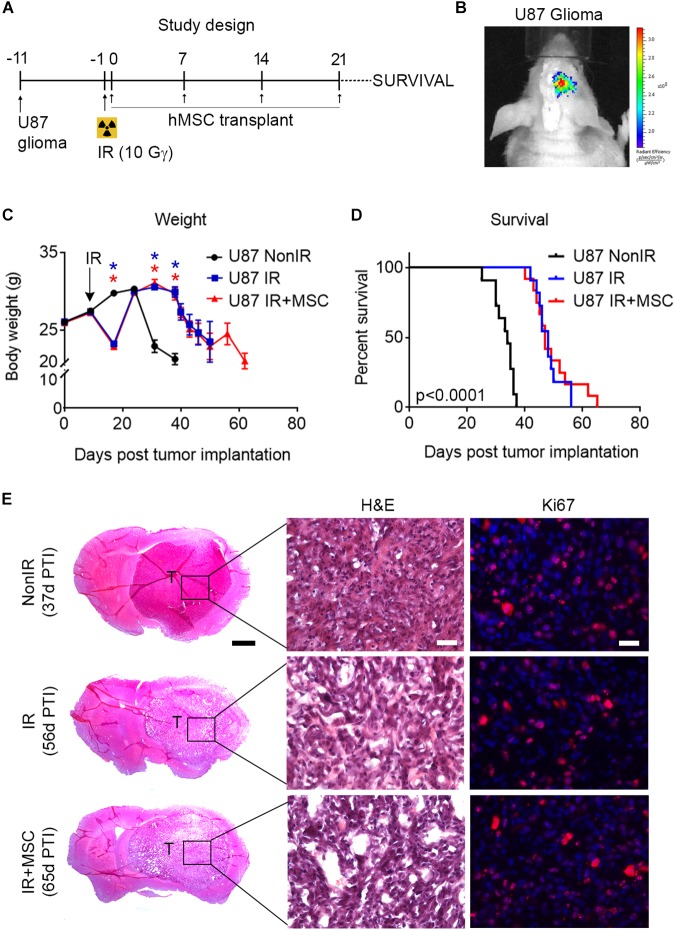
hMSC administration does not compromise survival of glioma-bearing mice. **(A)** Schematic representation of the study design. U87 glioma cells were intracranially transplanted into the striatum of nude mice. After 10 days, mice received whole-brain radiation (total dose of 10 Gy) and the day after, 5 × 10^5^ hMSCs were administered intranasally once per week for 4 weeks and time of death was monitored. **(B)** Xenolight DiR-labeled U87 glioma cells were locally transplanted into the striatum of nude mice. Images show bioluminescence activity in a representative animal 24 h after cell transplantation. **(C)** Animal weight was measured during the course of the experiment. Note the weight loss after radiation exposure. **(D)** Kaplan–Meier curve showing the percentage of survival of glioma-bearing mice. Note that IR and IR+MSC mice exhibited similar response, increasing survival as compared to NonIR mice. **(E)** Histological images of brain tumors at the time of death (indicated as days post tumor implantation, PTI) in the last individual surviving for each experimental group. H&E: Hematoxylin and eosin staining. Scale bar 1 mm (25 μm for details). Data are represented as mean ± SEM. *n* = 11–12 per group. ^∗^*p* < 0.0001 compared to U87 NonIR mice; Multiple *t*-test **(C)**, Log-rank test **(D)**.

Finally, we aimed to evaluate whether intranasal delivery of hMSCs affects life expectancy of brain tumor-bearing mice. For this, U87 glioma cells were intracranially implanted into the right striatum of athymic nude mice. Ten days post-glioma implantation mice received whole-brain radiation and the following day intranasal administration of hMSCs was initiated (a dose of cells per week during 4 consecutive weeks) (Figure 6A,B). All mice exposed to radiation lose ∼15–20% of body weight on the week after irradiation. However, body weight loss was rapidly recovered, reaching values of non-irradiated animal (U87 NonIR) by 2-weeks post-irradiation. During the course of the experiment weight loss prior to animal death was also observed in all experimental groups (Figure 6C). Irradiated glioma-bearing mice (i.e., U87 IR and U87 IR+MSC) exhibited a ∼40% extended median survival when compared to U87 NonIR mice. Despite median survival showed no significant differences between U87 IR and U87 IR+MSC, maximal longevity was registered in U87 IR+MSC mice (Figure 6D). Histopathological analysis of the brain at the time of death suggested no differences in tumor progression between groups, as evidenced by hematoxylin and eosin staining and immunohistochemistry against Ki67 (Figure 6E). These findings indicate that hMSC treatment does not compromise survival of mice after oncological radiotherapy.

## Discussion

The life expectancy of cancer patients has increased over the past 10 years due to more effective treatments. However, the greater effectiveness of these treatments is commonly associated with a high cost, since patients often face late severe side effects that significantly limit their quality of life. The majority of brain tumor patients that receive cranial radiation exhibit cognitive dysfunction that includes deficits in learning, memory, language, attention, and executive function (Ali et al., 2018). Here we demonstrate that the intranasal delivery of hMSCs prevents late neurofunctional sequelae after radiotherapy in mice. Our results hold promise in the prevention of radiation-induced damages in oncological patients to maximize their quality of life, particularly in pediatric patients whose developing brain is more radiosensitive.

Seminal reports have shown promising results using stem cells to prevent radiation-induced damages, although the cells were intracerebrally or systemically administrated (Acharya et al., 2009, 2011; Joo et al., 2012; Piao et al., 2015). The intranasal delivery proposed here is a clinically relevant strategy due to different aspects. First, the intranasal route is a non-invasive and feasible method of cell delivery that allows the transplantation of multiple doses of cells, as compared to intracranial implantation. Second, therapeutic effectiveness of systemic administration is hampered by the blood–brain barrier (BBB) (Li et al., 2015), while the intranasal delivery provides a practical method that efficiently bypasses the BBB allowing transplanted cells to reach the brain within minutes to rapidly accomplish their therapeutic effects (Danielyan et al., 2009). In addition, nose-to-brain cell delivery uses the olfactory and trigeminal extracellular pathways to distribute the cells throughout the central nervous system, thus eliciting effects at multiple sites within the brain (Thorne et al., 2004; Danielyan et al., 2009). Accordingly, we found that intranasal administration of hMSCs improves motor coordination, olfaction and memory, which are functions coordinated by specific areas located in different parts of the brain. This represents an advantage over intracerebral administration, which requires injections at different sites of the brain to achieve multiple neurofunctional effects. In this regard, a previous study demonstrated that bilateral injections of hOPCs into the corpus callosum of rats prevent memory deficits after radiation, while concomitant transplantation of hOPCs into the cerebellum was necessary to obtain benefits on motor function (Piao et al., 2015). Finally, our model focuses on the use of hMSCs derived from the adipose tissue, which represents an interesting clinical option due to the ease of obtaining large quantities of cells using a minimally invasive procedure (Lescaudron et al., 2012; Escacena et al., 2015).

Radiation injury has a multifactorial etiology that includes vascular damage (Li et al., 2003; Brown et al., 2005), demyelination (Panagiotakos et al., 2007; Gazdzinski et al., 2012), inflammation (Hwang et al., 2006), neuronal death, and neurogenesis decline (Achanta et al., 2012; Capilla-Gonzalez et al., 2014, 2016). In particular, inflammatory processes have been proposed as a major triggering factor leading to neuropathology in the irradiated brain (Acharya et al., 2016). In this regard, MSCs are known to exhibit immunomodulatory and anti-inflammatory properties (Hmadcha et al., 2009; Prockop and Oh, 2012; Escacena et al., 2015; Fu et al., 2017). Therefore, MSCs might be the ideal candidate for restoring brain homeostasis in radiation-induced inflammatory milieu. Here, we found that the therapeutic application of hMSCs reduces neuroinflammation and oxidative damage in whole-brain irradiated mice, which may help to prevent the loss of mature neurons. In contrast, newly born neurons were not protected by the hMSC treatment. The study of the neurogenic niche after a longer post-treatment period of time would be of interest to conclusively determine if intranasal hMSC delivery rescues irradiation-induced damages in neurogenesis. Consistently with the histopathological study, gene expression profiling at long-term evidences a modulation of neuroprotective processes, such those induced by melatonin (Mendivil-Perez et al., 2017; Paul et al., 2018), and changes in immune response pathways in the PLv and the Hipp of IR+MSC mice. Remarkably, we found a robust long-term activation of CREB signaling in irradiated mice, which was attenuated in mice receiving hMSC. In response to injury, CREB coordinates expression of genes encoding neuroprotective processes, such as BDNF that ultimately reduces inflammation, prevents oxidative damage and promotes anti-apoptotic effects (Han et al., 2000; Han and Holtzman, 2000). We also observed that both ERK and GSK3β signaling pathways play an important role in mediating CREB activation. The overall interpretation of our data indicates that hMSC transplantation allows a rapid and efficient repair of radiation-induced damages in mice, which is reflected in the normalization of neuroprotective cellular pathways at long-term post-radiation (i.e., 50 days post-radiation). However, further studies are required to fully understand the specific mechanisms that hMSCs modulate to induce radiation-damages repair. Based on our results and previous studies, we speculate that the beneficial effects of hMSCs are due to their paracrine activity rather than neural differentiation of hMSCs (Li et al., 2005; Wakabayashi et al., 2010; Wu et al., 2018). Extracellular vesicles (EVs) released by hMSCs are one of the main actors involved in the paracrine effects by modulating processes such as immune responses, inflammation, angiogenesis and homeostasis maintenance (Keshtkar et al., 2018). Indeed, latest research on regenerative medicine are moving toward cell-free therapies by employing stem cell-derived EVs due to their reduced immunogenicity (Zhu et al., 2017). However, obtaining sufficient amounts of EVs is still a major challenge toward their clinical application.

The therapeutic application of hMSCs to reduce neurological complications of radiation could be debatable due to their potential implications in tumor progression (Shahar et al., 2017; Whiteside, 2018). However, our pre-clinical study and others demonstrate that hMSC treatment does not compromise the survival of rodents with brain tumors (Balyasnikova et al., 2014; Li et al., 2014; Pacioni et al., 2017). Indeed, beneficial effects of hMSC administration have been reported through inhibition of tumor growth in orthotopic glioblastoma xenografts (Pacioni et al., 2017). Furthermore, the therapeutic effect of hMSCs to ameliorate complications associated with cancer treatments is currently under evaluation in humans. In this line, a Phase I clinical trial is recruiting patients to evaluate the safety and feasibility of delivering hMSCs by intracardiac injections to cancer survivors with cardiomyopathy induced by the chemotherapeutic anthracycline (NCT02509156). Another Phase II clinical study is also evaluating the safety and feasibility of hMSC injections into the submandibular gland to revert radiation-induced xerostomia in head and neck cancer patients (NCT02513238).

In conclusion, we demonstrate that intranasally delivered hMSCs is a non-invasive and effective treatment to promote brain damage repair after radiation and to improve neurological function in mice. These results also hold great promise for other inflammatory disorders, as well as for those diseases involving cognitive deterioration. The robust pre-clinical data presented here encourages the clinical use of hMSCs in cell-based therapy as an attractive option to prevent side effects induced by oncological radiotherapy. Despite the therapeutic window after radiation should be defined in brain tumor patients, hMSC application to reverse normal tissue toxicity could become an essential step in their treatment schedule.

## Ethics Statement

All animal handling procedures were approved by the CABIMER Ethics Committee for Animal Experimentation, and complied with national and European Union legislation (Spanish RD 53/2013 and EU Directive 2010/63) for the protection of animals used for scientific purposes.

## Author Contributions

BS, AM-M, AH, and VC-G contributed to the conceptualization, methodology and funding acquisition. AM-M, YA, NM-D, JL-B, MA-D, and VC-G performed the investigation. AM-M and VC-G wrote the original draft. BS, AM-M, EL, JAB, AH, and VC-G wrote, reviewed and edited the manuscript. BS, IH-H, EL, JAB, AH, and VC-G provided the resources. VC-G supervised the study.

## Conflict of Interest Statement

The authors declare that the research was conducted in the absence of any commercial or financial relationships that could be construed as a potential conflict of interest.
