# Supplementary material for: Human Mesenchymal Stem Cells Prevent Neurological Complications of Radiotherapy
Source: Front Cell Neurosci. 2019 May 16;13:204. doi: 10.3389/fncel.2019.00204 (PMC6532528; doi:10.3389/fncel.2019.00204)
Supplement: Supplementary file 1 [file Data_Sheet_1.PDF]

Supplementary Material

Supplementary Figures and Tables

This document includes 5 Supplementary Figures and 3 Supplementary Tables

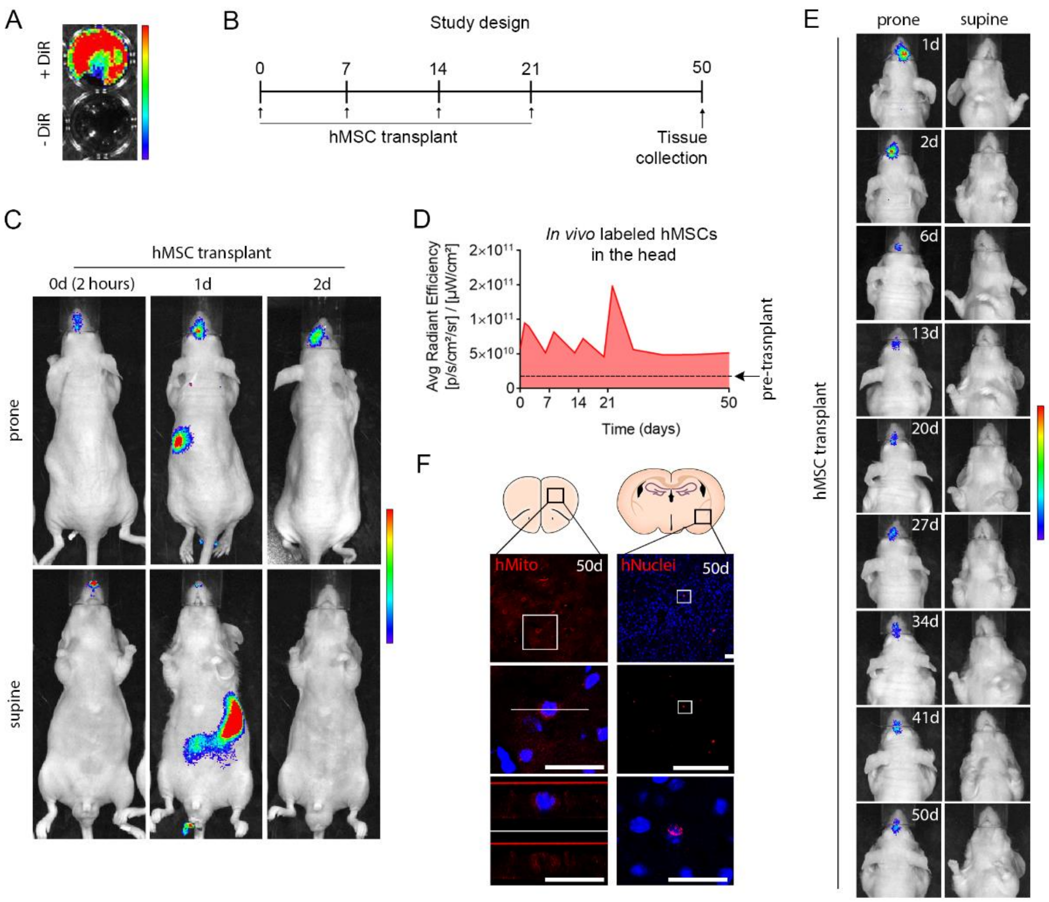

Supplementary Figure 1. hMSCs reached the brain after nasal administration. (A) Prior cell transplant, cultured hMSCs were labeled with the Xenolight DiR. (B) Schematic representation of the study design. Xenolight DiR-labeled hMSCs were administrated into the nasal cavity of athymic nude mice once per week for 4 weeks ($5{\cdot}10^5$ cells per week). Then, mice were sacrificed at day 50. (C) Representative images showing *in vivo* fluorescence signal of intranasally transplanted hMSCs at 2 hours, 1 day and 2 days post transplantation. (D) Quantification of the *in vivo* fluorescence signal in the head over time. (E) *In vivo* fluorescence signal of transplanted cells examined over time (up to 50 days). (F) Immunofluorescence against hMito and hNuclei revealed the presence of hMSCs 50 days after intranasal cell administration. Scale bar F: 25 µm. Rainbow color scale: red indicates highest fluorescence signal and blue indicates lowest fluorescence signal. n=3 per group.

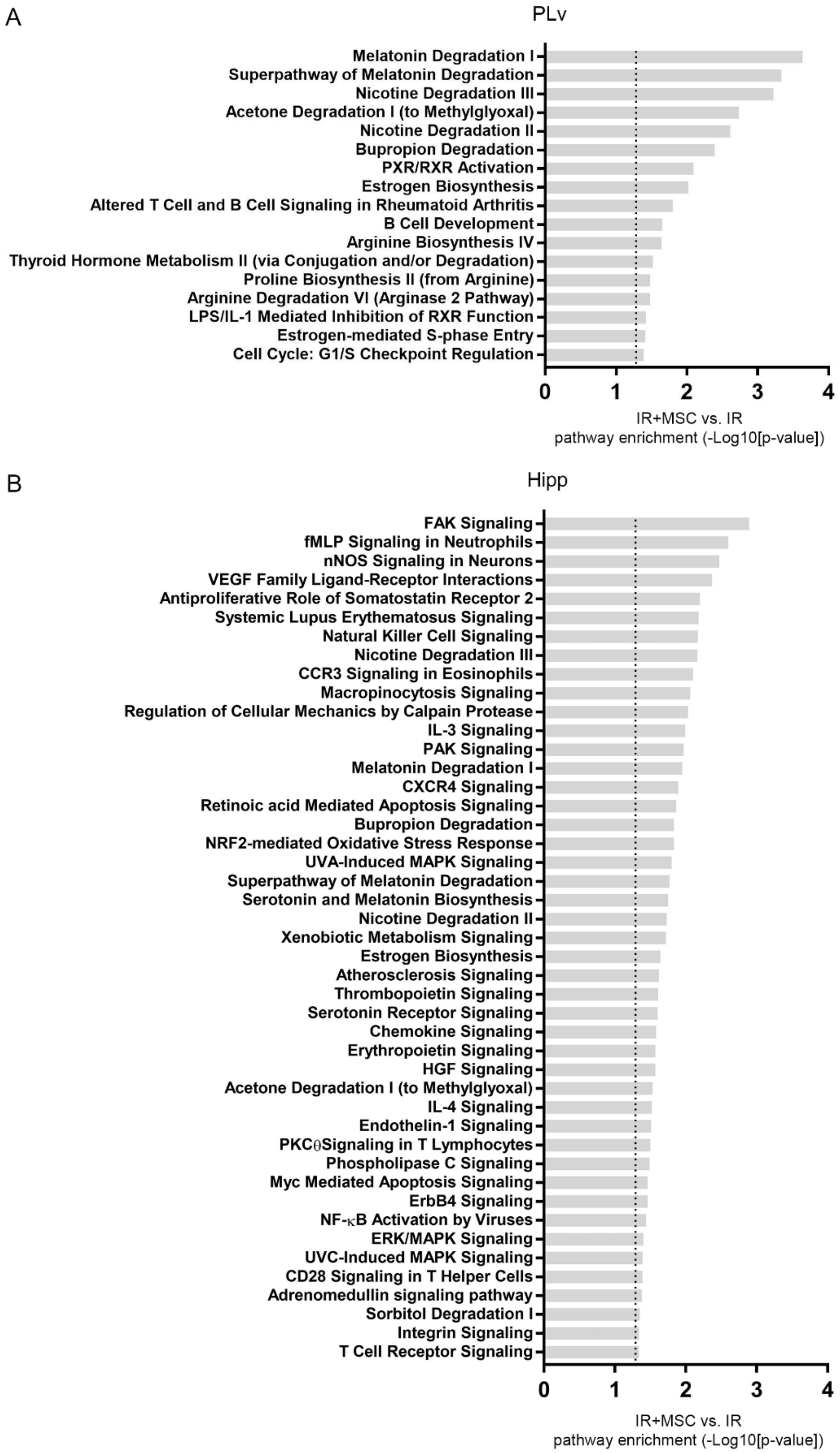

Supplementary Figure 2. Significantly modulated canonical pathways by IPA. (A) Significantly modulated canonical pathways in the PLv of IR+MSC vs. IR. (B) Significantly modulated canonical pathways in the Hipp of IR+MSC vs. IR. n=3 per group.

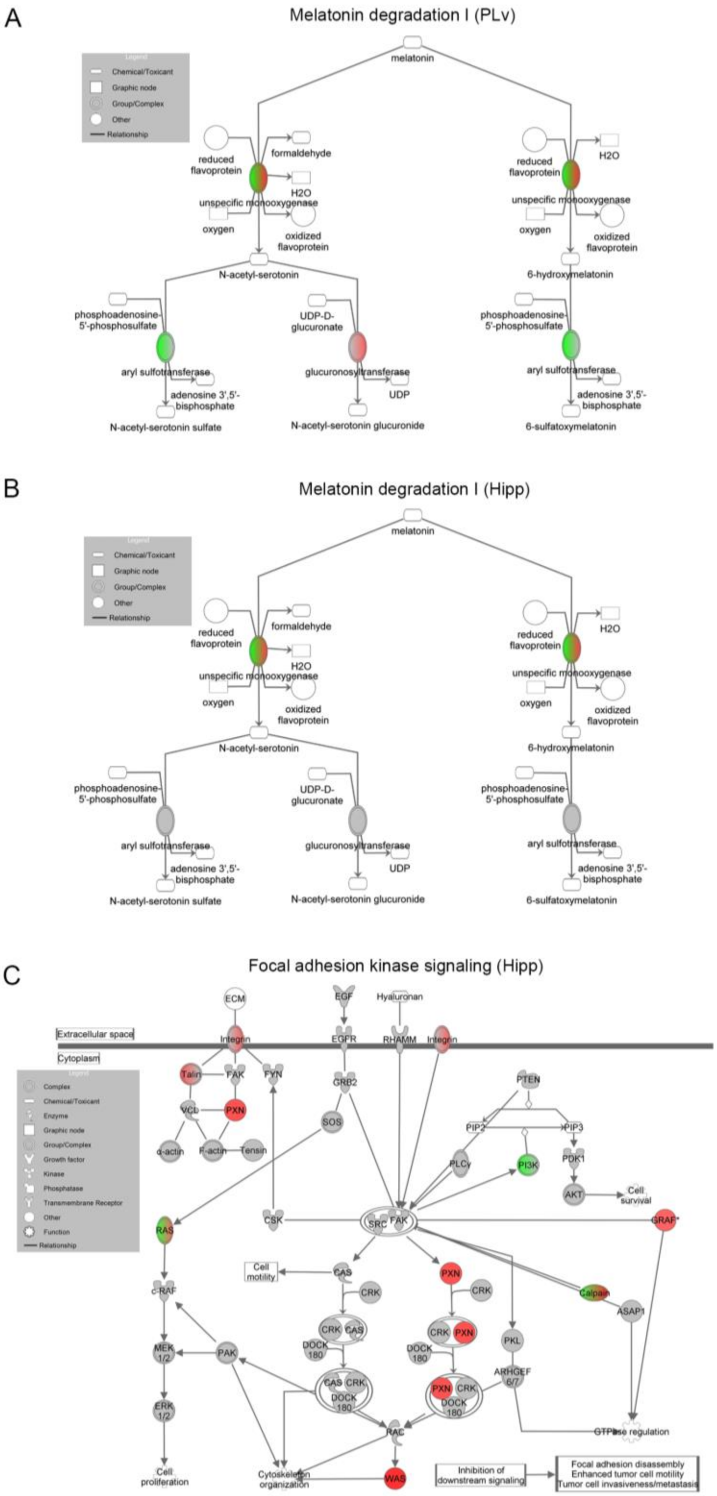

Supplementary Figure 3. Representative images of significantly modulated canonical pathways in the comparison IR+MSC vs. IR. (A) Representative IPA image of “melatonin degradation I” pathway in the PLv of MSC+IR vs. IR. (B) Representative IPA image of “melatonin degradation I” pathway in the Hipp of MSC+IR vs. IR. (C) Representative IPA image of “Focal adhesion kinase signaling” pathway in the PLv of MSC+IR vs. IR.

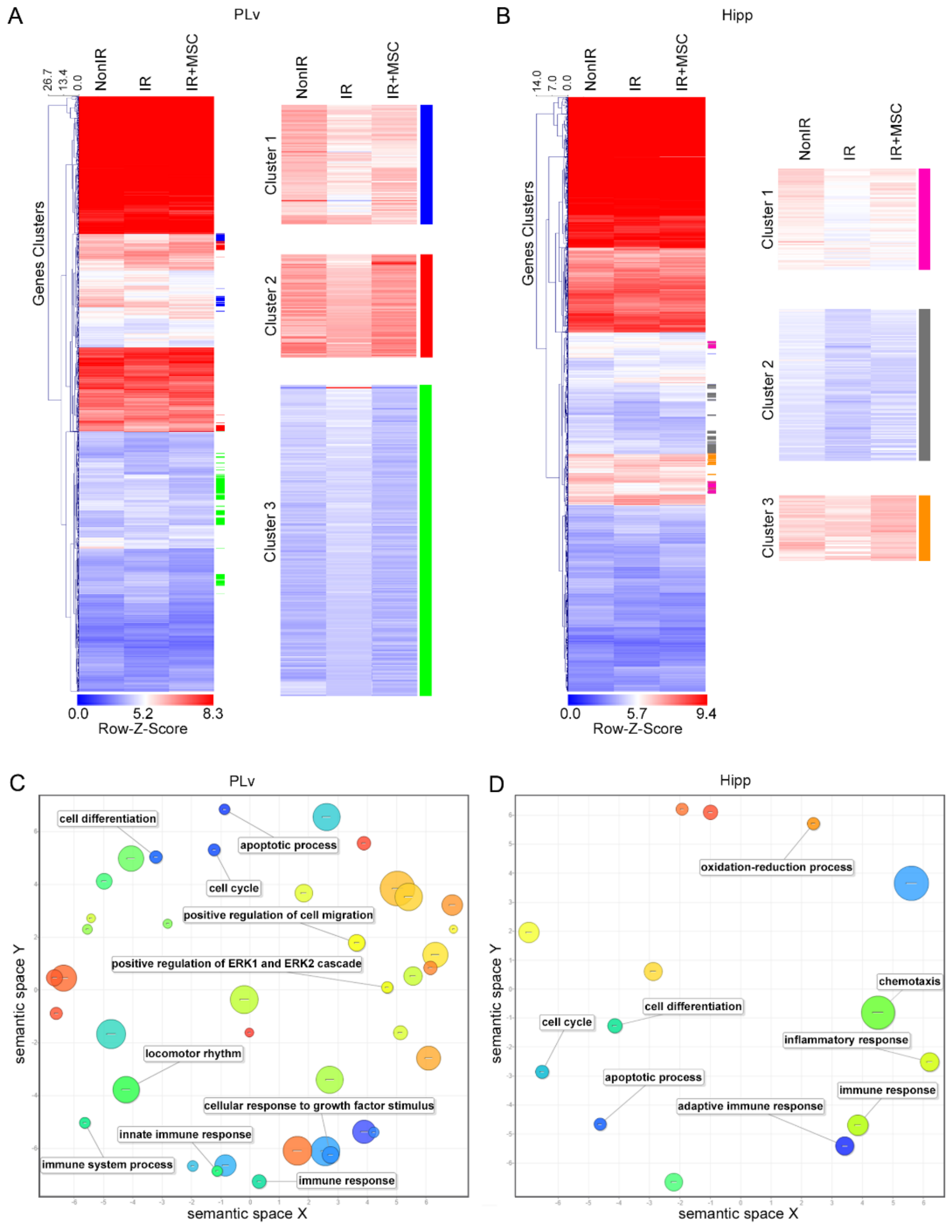

Supplementary Figure 4. The gene expression profile of the irradiated brain was modulated by hMSC transplantation. (A) Hierarchical clustering of genes differentially expressed in the PLv for at least one comparison (Non-IR vs. IR, Non-IR vs. IR+MSC and IR vs. IR+MSC). The three selected clusters represent genes with similar transcriptional profiles in the comparison Non-IR vs. IR+MSC. Red indicates high expression and blue indicates low expression. (B) Hierarchical clustering of genes differentially expressed in the Hipp for at least one comparison (Non-IR vs. IR, Non-IR vs. IR+MSC and IR vs. IR+MSC). The three selected clusters represent genes with similar transcriptional profiles in the comparison Non-IR vs. IR+MSC. Red indicates high expression and blue indicates low expression. (C) Scatterplot of GO analysis from the three selected clusters in A showing the most enriched biological processes (enrichment score ≥ 1.0). Bubble size indicates *p*-value (-log10 *p*-value). (D) Scatterplot of GO analysis from the three selected clusters in B showing the most enriched biological processes (enrichment score ≥ 1.0). Bubble size indicates *p*-value (-log10 *p*-value). Hierarchical clustering of genes (average linkage and Euclidean distance as similarity measure) was performed by K-mean clustering (KMC) algorithm with the open-source data analysis Multiple

Experiment Viewer (MeV) software package v.4.9.0. DAVID online bioinformatics resource v6.8 was used for GO analysis. Scatterplots of GO analysis were generated using the open-source online tool REVIGO.

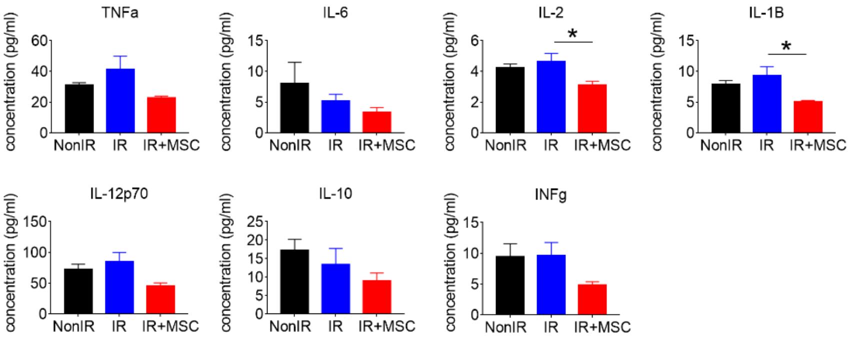

Supplementary Figure 5. Plasma levels of inflammatory cytokines. Concentrations of inflammatory cytokines were determined in plasma using Bio-Plex Pro Mouse Cytokine Assay (Bio-Rad Laboratories). Blood samples were collected from tail vein puncture on day 50 post-radiation in order to obtain the plasma. Data are represented as mean ± SEM. n=4-5 per group *p<0.05, One-way ANOVA.

Supplementary Table 1. List of reagents and resources used in this study.

REAGENT or RESOURCE	SOURCE	IDENTIFIER
**Antibodies**		
Lys-4-HNE (used at 1:1000)	Merk Millipore	Cat # 393206, RRID:AB_211975
AKT (used at 1:1000)	Cell Signaling	Cat # 9272, RRID:AB_329827
BDNF (used at 1:1000)	StressMarq Biosciences	Cat # spc703, RRID:AB_2570711
Caspase 3 (used at 1:1000)	Cell Signaling	Cat # 9662, RRID:AB_331439
CBP (used at 1:100)	Santa Cruz Biotechnology	Cat # sc369, RRID:AB_631006
CD68 (used at 1:150)	BioRad	Cat # MCA1957GA, RRID:AB_324217
CREB (used at 1:1000)	Cell Signaling	Cat # 9197, RRID:AB_331277
DCX (used at 1:200)	Santa Cruz Biotechnology	Cat # sc8066, RRID:AB_2088494
ERK 1/2 (used at 1:2000)	Cell Signaling	Cat # 9102, RRID:AB_330744
GAPDH (used at 1:1000)	Cell Signaling	Cat # 2118, RRID:AB_561053
GFAP (used at 1:500)	Merk Millipore	Cat # MAB360, RRID:AB_11212597
GSK3β (used at 1:200)	Santa Cruz Biotechnology	Cat # sc9166, RRID:AB_647604
Human mitocondria (used at 1:100)	Merk Millipore	Cat # MAB1273, RRID:AB_94052
Human nuclei (used at 1:200)	Merk Millipore	Cat # MAB1281, RRID:AB_94090
Iba 1 (used at 1:1000)	Wako	Cat # 019-19741, RRID:AB_839504
iNOS (used at 1:100)	Merk Millipore	Cat # ABN26, RRID:AB_10805939
Ki67 (used at 1:200)	Fisher Scientific	Cat # RM-9106-S1, RRID:AB_149792
Nestin (used at 1:100)	BD Biosciences	Cat # 556309, RRID:AB_396354
NeuN (used at 1:700)	Merk Millipore	Cat # MAB377, RRID:AB_2298772

pSer473 AKT (used at 1:500)	Cell Signaling	Cat # 9271, RRID:AB_329825
pSer133 CREB (used at 1:1000)	Cell Signaling	Cat # 9198, RRID:AB_2561044
pThr202/Tyr204 ERK 1/2 (used at 1:2000)	Cell Signaling	Cat # 9106, RRID:AB_331768
pSer9 GSK3β (used at 1:500)	Cell Signaling	Cat # 9336, RRID:AB_331405
PI3K (used at 1:1000)	Cell Signaling	Cat # 4292, RRID:AB_329869
pTyr199 PI3K (used at 1:1000)	Cell Signaling	Cat # 4228, RRID:AB_659940
**Experimental Models: Cell Lines**		
PCS-500-011	ATCC	https://www.lgcstandards-atcc.org/Products/All/PCS-500-011
U-87 MG	ATCC	https://www.lgcstandards-atcc.org/products/all/HTB-14

Supplementary Table 2. List of the 20 most up-regulated and down-regulated significantly modulated genes in PLv. n=3 per group.

**IR vs NonIR**			**IR+MSC vs NonIR**			**IR+MSC vs. IR**		
Gene Symbol	***Fold Change***	***P-value***	***Gene Symbol***	***Fold Change***	***P-value***	***Gene Symbol***	***Fold Change***	***P-value***
Gm17482	57,41	0,0094	Map2	25,93	0,0199	Kansl1l	6,72	0,0041
Acvr1c	17,74	0,0192	Kansl1l	5,45	0,0013	Calb2	3,72	0,0063
Gm8140	6,4	0,0172	Tmem68	4,19	0,0128	Resp18	3,19	0,0042
Tmem68	4,33	0,0131	Gpr101	3,78	0,0214	Npsr1	2,94	0,0035
Olfr1058	3,51	0,0264	F830001A07 Rik	3,58	0,0002	4932438H23R ik	2,94	0,0093
Inhba	3,34	0,0399	Clic6	3,39	0,0015	Cd46	2,81	0,0003
F830001A07 Rik	2,92	0,0002	Resp18	3,23	0,0135	Ptgfr	2,77	0,0049
Gm11114	2,84	0,0135	Aox1	2,74	0,0125	Kif11	2,65	0,02
Olfr1180	2,77	0,0146	Ephx1	2,62	0,0002	Cxcl13	2,47	0,0486
Gm6654	2,76	0,0038	Ebf4	2,6	0,0059	Avp	2,44	0,0235
Il15	2,76	0,0054	Coa4	2,57	0,0029	LOC1008616 15; Gm3411	2,42	0,0241
Olfr273	2,76	0,0092	Ifit3b	2,52	0,0009	Gm14308	2,41	0,0001
Cd180	2,69	0,0017	Olfr266	2,47	5,51E-05	Gm14308; Gm14430; Gm14432; Gm4724; 0610010B08R ik	2,36	1,26E-05
Eda2r	2,57	0,0415	Ogn	2,45	0,0346	Mad2l2	2,31	0,0046
Glipr1	2,55	0,0006	Trim30a	2,35	0,0013	Lgals3bp	2,29	0,0002
Vmn1r228	2,47	0,0039	Olfr421-ps1	2,35	0,0095	Pvrl1	2,29	0,0469
Tcea3	2,46	0,0021	Masp2	2,35	0,0146	Mcam	2,25	0,018
Gm20773	2,45	0,0386	Prrg4	2,34	0,0044	Gm14444	2,23	0,0026
Gm20851	2,45	0,0386	Clec2d	2,3	0,021	Gm14406	2,23	0,0026
Olfr867	2,43	0,0055	Stard6	2,29	0,0023	Olfr1273-ps	2,23	0,0371
Adgrf1	-2,66	0,0036	Pdk4	-2,7	0,0005	Gm11037	-2,33	0,0026
Depdc1a	-2,72	0,0059	Hist1h2ak	-2,75	7,46E-05	Gm5726; Gm8677	-2,37	0,02

Calb2	-2,74	0,0239	Neurod2	-2,77	0,0273	Gm5726; Gm8677	-2,37	0,02
Dlx1	-2,77	0,0054	Mup13; Mup18; Mup16; Mup14; Mup17; Mup2; Mup15	-2,81	0,0004	Igfbp6	-2,39	0,0244
Slc6a7	-2,77	0,0276	Mfap2	-2,88	0,0014	Nox3	-2,39	0,0425
Gm3099	-2,81	0,0382	LOC100040054	-2,9	0,0146	Gm4687	-2,4	0,0059
Pdk4	-2,82	0,0009	Hmgb2	-2,92	0,0002	Olfr390	-2,41	1,57E-05
Hist1h2ak	-2,89	0,0003	Hist2h3b	-2,93	0,0015	Acss3	-2,42	0,0103
Ccdc18	-2,91	0,0067	Gm14525	-3,03	0,0049	LOC100503047; Gm10264	-2,44	0,0047
Kif11	-3,14	0,0056	Mylpf	-3,05	0,0304	Fam132b	-2,48	0,0061
Txnip	-3,14	0,0289	Fezf2	-3,16	0,0128	Gm21860	-2,6	0,0313
Hmgb2	-3,24	0,0001	Gm21699	-3,39	0,0077	Gm21748	-2,6	0,0313
Tmem252	-3,3	0,004	Hist2h3c2	-3,58	0,0009	Fpr1	-2,62	0,0264
Npsr1	-3,39	0,0005	Hist2h3c1	-3,58	0,0009	Olfr491	-2,74	0,0071
Dlx2	-3,83	0,0003	Dlx2	-3,71	0,0012	Olfr1049	-2,92	0,0069
Hist2h3c2	-3,88	0,0005	Ccdc7b	-3,74	0,0039	Gm6654	-3,04	0,0016
Hist2h3c1	-3,88	0,0005	Gm21608	-3,85	2,30E-05	Fstl1	-3,21	0,0474
Mfap2	-5,37	4,40E-05	Serinc2	-3,88	0,0071	Vmn1r228	-3,65	0,0009
Pbk	-6,31	6,97E-06	Tmem252	-4,14	0,0196	Gm14525	-3,7	0,0022
Gm11096	-13,72	0,0042	Pbk	-5,3	9,00E-06	Gm17482	-55,21	0,0041

Supplementary Table 3. List of the 20 most up-regulated and down-regulated significantly modulated genes in Hipp. n=3 per group.

**IR vs NonIR**			**IR+MSC vs NonIR**			**IR+MSC vs. IR**		
Gene Symbol	***Fold Change***	***P-value***	***Gene Symbol***	***Fold Change***	***P-value***	***Gene Symbol***	***Fold Change***	***P-value***
Map2	21,84	0,0369	Map2	21,81	0,0123	Tgtp2	3,79	0,0281
Fmod	11,77	0,0073	Kansl1l	7,16	0,0054	Gm3893; 4933409K07Rik	3,26	0,0121
Ptgds	5,74	0,0014	Sema5a	3,78	0,0375	Aox1	3,1	0,0003
Ecel1	5,29	0,0198	Cxcl13	3,63	0,0152	Gm7120	2,92	6,44E-05
Zic1	5,03	0,0013	Ifit3	3,56	0,0004	Capn3	2,91	0,0087
Aebp1	4,07	0,0078	Zic1	3,36	0,0013	Gm7682	2,81	0,001
Arhgap6	3,83	0,0209	Cybb	3,21	0,0176	Gm20823	2,71	0,0138
Ntsr1	3,6	0,0137	Otx2	3,06	0,0464	Gm20738; Gm20823	2,68	0,0057
Cox6b2	3,55	0,0004	Gpr50	2,97	0,0203	Ush1c	2,64	0,0413
Slc13a4	3,49	0,0045	Glra3	2,93	0,0464	Armcx1	2,57	0,0014
Gm11077	3,39	0,0125	Pqlc3	2,92	0,0485	Igf2bp1; Mir3063	2,51	0,0018
Chst9	3,35	0,0058	Zfp119b	2,85	0,0003	Vmn1r28	2,5	0,0412
Slc6a20a	3,22	0,0079	Capn3	2,72	0,0348	Hesx1	2,44	0,046
Slc22a6	3,13	0,0007	Cd74; Mir5107	2,65	0,0447	Gm20331	2,38	0,0028
Mgp	3,1	0,0231	Aox1	2,61	0,0018	Olfr914	2,34	0,0075
Slc6a13	2,94	0,0018	Dsp	2,61	0,0043	Eif2s3y	2,33	3,19E-05
Ogn	2,83	0,0046	Adcyap1	2,61	0,0371	Kif4	2,33	0,0017
Slc22a2	2,82	0,0256	Mup2; Mup1	2,56	0,0064	Psg25	2,3	0,0033
Camk2d	2,79	0,0053	Cox6b2	2,55	0,0019	Nt5dc3	2,3	0,0076
Chrdl1	2,74	0,0038	Slc6a13	2,53	0,0403	Trim30a	2,28	0,0011
Vmn1r21	-2,49	0,0492	Tbata	-2,18	0,0015	Armcx6	-2,08	0,0045
Ccl19	-2,54	0,0009	Kir3dl2	-2,21	0,0087	Zcchc10	-2,15	0,0068
Fos	-2,54	0,0116	C130026I21Rik	-2,23	0,0379	Olfr807	-2,16	0,0204
Olfr914	-2,59	0,0035	Pvr	-2,23	0,0416	Pla2g4e	-2,16	0,048

Gm3893; 4933409K07Rik	-2,61	0,0374	Tnfaip3	-2,28	0,0003	Kcnk5	-2,2	0,0019
Spink8	-2,64	0,0351	4930548G14Rik	-2,3	0,0002	Cox18	-2,23	0,0006
Lrriq1	-2,7	0,0476	Vmn1r57	-2,33	0,0276	Cytl1	-2,25	0,0072
Arl4d	-2,71	7,60E-05	Dap	-2,42	0,0301	Nmur1	-2,27	0,0134
Strip2	-2,71	0,0296	AI506816	-2,45	0,0368	Pdcl2	-2,39	0,0086
Olfr207; Olfr208; Olfr209	-2,72	0,0246	Gm26727	-2,48	0,0024	Slc22a2	-2,41	0,0459
Vmn1r57	-2,79	0,0143	Olfr207; Olfr208; Olfr209	-2,54	0,0199	Rbm43	-2,56	0,0408
Armcx1	-2,84	0,0007	A530053G22Rik	-2,65	0,0258	Gm10715	-2,63	0,0044
Mfsd2a	-2,84	0,0066	Mfsd2a	-2,71	0,0067	Vmn2r45	-2,65	0,0175
Eps8l1	-2,93	0,0028	Olfr623	-2,72	0,0164	Gm17535	-2,71	0,004
Keg1	-2,95	0,0109	Nr4a1	-2,96	0,0111	Gm3317	-2,76	0,0081
Tnfaip3	-3,26	2,45E-05	Fosb	-3,11	0,0005	Gm3488; Gm3317	-2,76	0,0081
Csf1r	-3,29	0,0005	Vmn1r21	-3,18	0,0013	Gm11115	-2,76	0,0133
Pccb	-3,74	0,0119	Gm10944	-3,38	0,0092	Gm3468	-3,15	0,0002
Nr4a1	-3,94	0,0148	Htr3a	-4,98	0,0309	Mgp	-3,25	0,0165
Egr2	-4,13	0,0007	Myl1	-5,53	0,0041	Acvr1c	-6,84	0,0464
